# Diffuse Alveolar Hemorrhage Due to Macrolide-Refractory Mycoplasma pneumoniae Diagnosed by FilmArray Analysis of Bronchoalveolar Lavage Fluid

**DOI:** 10.7759/cureus.75525

**Published:** 2024-12-11

**Authors:** Tatsumi Nakashima, Toyoshi Yanagihara, Rei Sanai, Yuta Fujimoto, Akira Nakao, Yuki Shundo, Naoki Hamada, Noriyuki Ebi, Hiroyuki Inoue, Masaki Fujita

**Affiliations:** 1 Department of Respiratory Medicine, Fukuoka University Hospital, Fukuoka, JPN

**Keywords:** diffuse alveolar hemorrhage, fiberoptic bronchoscopy, filmarray respiratory panel 2.1, mycoplasma pneumoniae infection, respiratory failure

## Abstract

*Mycoplasma pneumoniae* typically causes mild respiratory infections but can rarely lead to severe complications. We report a case of a 43-year-old immunocompetent male who presented with progressive dyspnea and respiratory failure with bilateral pulmonary infiltrates, refractory to outpatient treatment with azithromycin, ceftriaxone, and levofloxacin. Bronchoscopy revealed multiple white plaques in the trachea and diffuse alveolar hemorrhage. Although initial throat loop-mediated isothermal amplification (LAMP) testing for *M. pneumoniae* was negative, FilmArray analysis of bronchoalveolar lavage fluid successfully detected *M. pneumoniae.* The patient improved with minocycline and methylprednisolone treatment. This case demonstrates that *M. pneumoniae* can cause severe complications, including diffuse alveolar hemorrhage, even in immunocompetent patients. In some cases, it highlights the importance of lower respiratory tract sampling for accurate diagnosis.

## Introduction

*Mycoplasma pneumoniae* (*M. pneumoniae*) is a common respiratory pathogen that typically causes mild upper respiratory tract infections and "walking pneumonia." It accounts for 4-8% of community-acquired pneumonia cases in endemic areas, increasing to up to 40% during epidemics [1]. While usually self-limiting, *M. pneumoniae *infection can occasionally lead to severe complications and extrapulmonary manifestations [1].

Diffuse alveolar hemorrhage (DAH) is a life-threatening syndrome characterized by bleeding into the alveolar spaces due to microvascular injury, with reported mortality rates of 20-100% [2]. Although DAH is most commonly associated with autoimmune conditions such as vasculitis, systemic lupus erythematosus (SLE), and rheumatoid arthritis [3], it can also occur in infectious diseases including influenza, dengue, leptospirosis, and rarely, *M. pneumoniae* infection [4]. Recent case reports have documented *M. pneumoniae*-associated DAH even in immunocompetent patients, demonstrating that this serious complication can develop despite appropriate antibiotic therapy [5,6].

Here we present a case of severe *M. pneumoniae* infection complicated by DAH in an immunocompetent adult, where the diagnosis was established through FilmArray analysis of bronchoalveolar lavage fluid (BALF) after initial negative throat loop-mediated isothermal amplification (LAMP) testing, highlighting the potential importance of lower respiratory tract sampling in such cases.

## Case presentation

**Figure 1:**
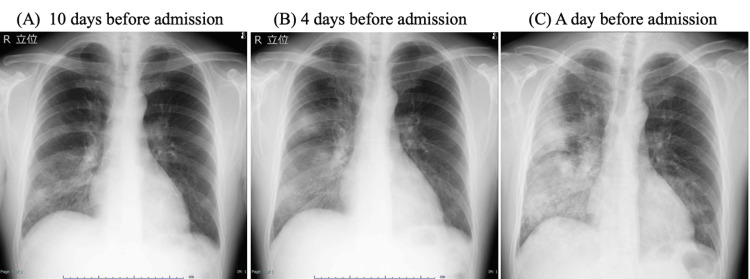
Serial chest X-ray imaging of the patient. (A) 10 days, (B) four days, and (C) one day before admission, showing progressive pulmonary infiltrates initially in the right lower lung, extending to the upper and left lung fields.

A 43-year-old previously healthy male presented to our hospital with progressive dyspnea. His past medical history was significant for atrial fibrillation treated with an ablation procedure. He had no ongoing anticoagulation or antiplatelet therapy. Ten days before admission, he developed high-grade fever (39°C), headache, and cough. He visited another hospital, where a SARS-CoV-2 antigen test was negative, but blood tests revealed elevated C-reactive protein (CRP) (26.4 mg/dL). Chest X-ray showed ground-glass opacity in the right lower lung field (Figure 1A). He was initially diagnosed with pneumonia and started on ceftriaxone 2 g/day and azithromycin 500 mg/day. The throat swab LAMP test for *Mycoplasma pneumoniae* was negative. Four days before admission, he revisited another hospital due to worsening symptoms. Chest X-ray revealed new infiltrates in the right lower lung field (Figure 1B), and his antibiotics were changed to levofloxacin 500 mg/day. The day before admission, further chest imaging showed expansion of right lung infiltrates and new ground-glass opacities in the left lower lung field (Figure 1C). He was referred to our department for further evaluation.

On the day of admission, he presented to our emergency department with acute worsening of dyspnea and oxygen desaturation (SpO_2_ <90%). On physical examination, his vital signs were: temperature 38.2°C, pulse 63/min (regular), blood pressure 123/68 mmHg, and respiratory rate 20/min. SpO_2_ was 85% while walking and 89% at rest on room air, requiring O_2_ supplementation with 3 L/min. Chest auscultation revealed bilateral coarse crackles predominantly in the lower lung fields, accompanied by productive cough. His right lower leg showed swelling, redness, and tenderness. His blood tests revealed an elevated CRP, AST, ALT, LDH, ALP, and D-dimer (Table 1).

**Table 1:** Blood test on admission. WBC: white blood cells; RBC: red blood cells; Hb: hemoglobin; Plt: platelets; TP: total protein; T-Bil: total bilirubin; AST: aspartate aminotransferase; ALT: alanine aminotransferase; LDH: lactate dehydrogenase; CK: creatine kinase; BUN: blood urea nitrogen; Cr: creatinine; Na: sodium; K: potassium; CRP: C-reactive protein; T-Chol: total cholesterol; TG: triglycerides; KL-6: Krebs von den Lungen-6; SP-D: surfactant protein D; BNP: brain natriuretic peptide; IgG: immunoglobulin G; IgM: immunoglobulin M; PT: prothrombin time; APTT: activated partial thromboplastin time; FDP: fibrin degradation products.

Test	Value	Reference range	Test	Value	Reference range
WBC (/uL)	8900	3300-8600	CRP (mg/dL)	18.06	<0.14
RBC (10^4^/μL)	411	435-555	T-Chol (mg/dL)	99	142-248
Hb (g/dL)	12.2	13.7-16.8	TG (mg/dL)	37	40-234
Plt (10^3^/uL)	384	158-348	KL-6 (U/mL)	535	<500
TP (g/dL)	7.6	6.6-8.1	SP-D (ng/mL)	565	<110
T-Bil (mg/dL)	0.8	0.4-1.5	BNP (pg/mL)	35.8	<18.4
AST (U/L)	52	13-30	IgG (mg/dL)	1443	861-1747
ALT (U/L)	54	10-42	IgM (mg/dL)	46	33-183
LDH (U/L)	303	124-222	Ferritin (ng/mL)	1191	39.9-465
CK (U/L)	44	59-248	PT (sec)	13.5	9.8-12.1
BUN (mg/dL)	9	8-20	APTT (sec)	25	24-34
Cr (mg/dL)	0.63	0.65-1.07	D-dimer (ug/mL)	112.4	<1.0
Na (mEq/L)	138	138-145	FDP (ug/mL)	314	<5.0
K (mEq/L)	3.6	3.6-4.8	Fibrinogen (mg/dL)	539	200-400

**Figure 2:**
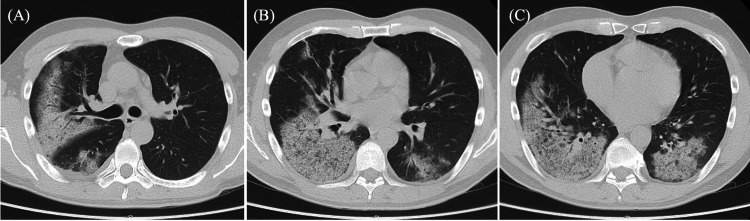
Chest CT imaging on admission. (A)–(C) Axial chest CT scans showing bilateral pulmonary infiltrates with consolidation and ground-glass opacities predominantly in the lower lung.

**Figure 3:**
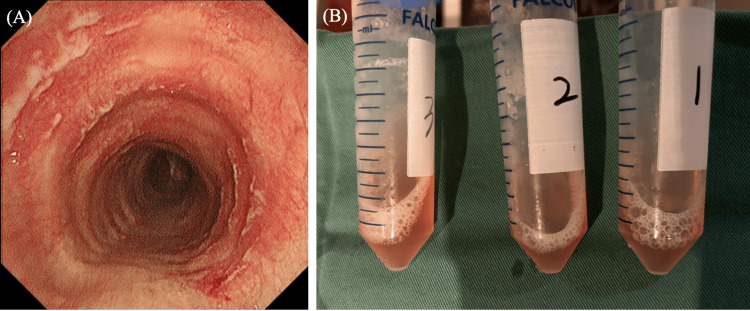
Bronchoscopy findings and bronchoalveolar lavage (BAL) fluid analysis. (A) Bronchoscopy showing white plaques and mild tracheal bleeding. (B) Serial BAL fluid samples displaying progressively hemorrhagic fluid, indicative of diffuse alveolar hemorrhage.

**Figure 4:**
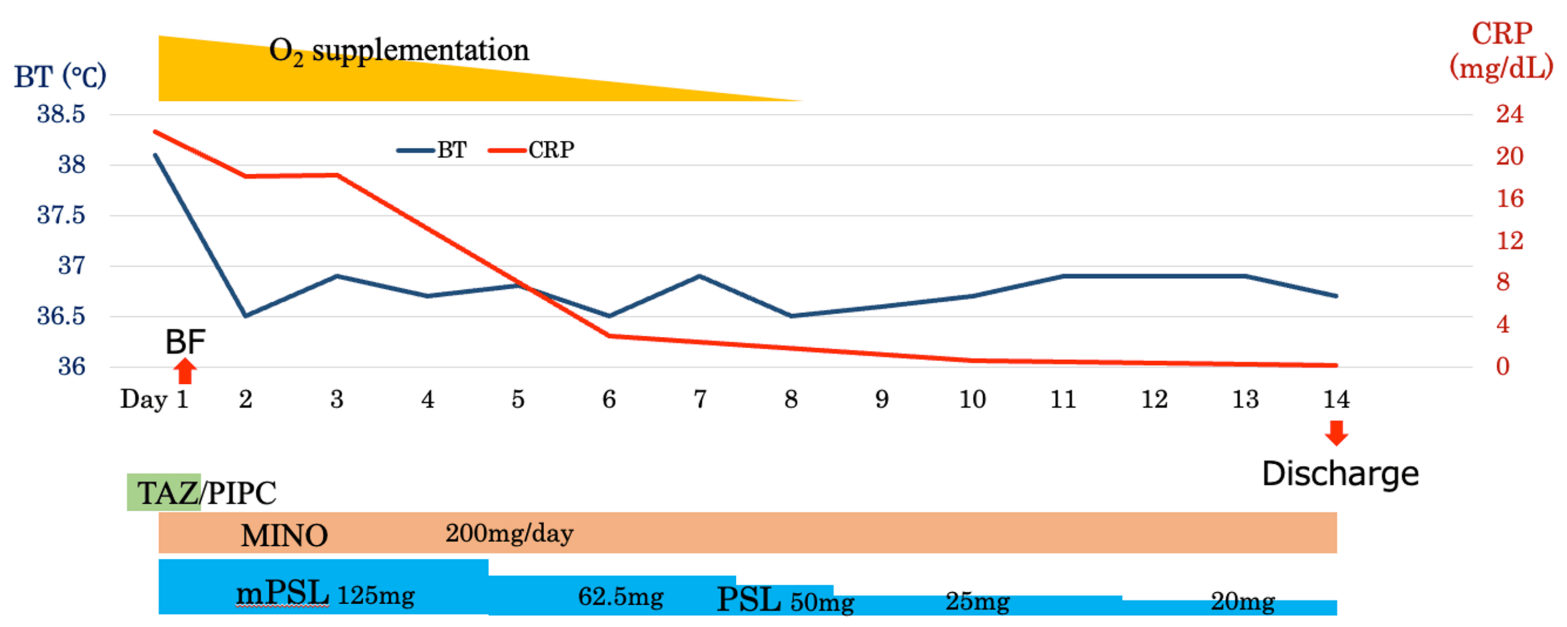
Clinical course and treatment timeline. Trends of body temperature (BT) and C-reactive protein (CRP) levels during hospitalization. The graph highlights the timeline of oxygen supplementation and pharmacological treatments, including TAZ/PIPC, minocycline (MINO), and corticosteroids (mPSL, PSL). Broncho-fiberscope (BF) was performed on day one, and the patient was discharged on day 14 after clinical improvement. TAZ/PIPC: tazobactam piperacillin, mPSL: methylprednisolone, PSL: prednisolone.

Chest CT imaging revealed bilateral consolidation predominantly in the lower lungs (Figure 2). Based on the acute clinical presentation of progressive antibiotics-refractory bilateral infiltrates with marked inflammation and respiratory failure, infectious pneumonia of unknown origin, alveolar hemorrhage, and organizing pneumonia were considered as differential diagnoses. Bronchoscopy was conducted promptly, which showed multiple white plaques with mild bleeding in the trachea (Figure 3A). Bronchoalveolar lavage (BAL) from the right B8 demonstrated progressively hemorrhagic return fluid, indicating alveolar hemorrhage (Figure 3B). BALF analysis showed lymphocytic predominance, and FilmArray Respiratory Panel 2.1 detected *M. pneumoniae*. Based on these findings, the patient was diagnosed with *M. pneumoniae* infection complicated by DAH and coagulopathy. Given the poor response to previous azithromycin and levofloxacin therapy, treatment was initiated with minocycline 200 mg/day. Additionally, methylprednisolone 125 mg/day was started for DAH with respiratory failure (Figure 4).

The patient became afebrile on day two of hospitalization, with a gradual improvement in symptoms. Oxygen supplementation was discontinued on day seven. As inflammatory markers improved, corticosteroid therapy was gradually tapered. Given the coagulopathy with marked elevation of D-dimer on admission, lower extremity venous ultrasonography was performed, revealing deep vein thrombosis. After improvement in oxygenation and resolution of alveolar hemorrhage, anticoagulation was initiated with heparin and later transitioned to oral anticoagulation. During the course, the HIV screening test was positive, raising concern for underlying immunodeficiency. However, subsequent HIV-RNA testing on day five was negative, suggesting a false-positive screening result (Table 2). Autoantibody screening was performed as part of the DAH workup. Although ANA titer was elevated at 1:160, no specific autoantibodies were detected. The *M. pneumoniae* antibody particle agglutination (PA) titer on admission was later found to be significantly elevated at 1:2560 (reference <1:320), further confirming *M. pneumoniae* infection (Table 2). The patient's condition continued to improve, and he was discharged home on day 14 of hospitalization.

**Table 2:** Additional blood test. CF: complement fixation; PA: particle agglutination; HTLV-1: human T-cell leukemia virus type 1; HIV: human immunodeficiency virus; RNA PCR: RNA polymerase chain reaction; CD: cluster of differentiation; MPO-ANCA: myeloperoxidase anti-neutrophil cytoplasmic antibody; PR3-ANCA: proteinase 3 anti-neutrophil cytoplasmic antibody; RF: rheumatoid factor; ANA: anti-nuclear antibody; SS-A: Sjögren's syndrome A (also known as Ro antibody); SS-B: Sjögren's syndrome B (also known as La antibody); CCP: cyclic citrullinated peptide; RNP: ribonucleoprotein; Sm: Smith; Scl-70: scleroderma-70; Pol III: RNA polymerase III.

Test	Value	Reference range	Test	Value	Reference range
Mycoplasma antibody (CF) (dil)	512	<4	RF (IU/mL)	6	<15
Mycoplasma antibody (PA) (dil)	2560	<40	ANA (dil)	160	<40
HTLV-1 antibody	–	Negative	SS-A antibody (U/mL)	<1.0	<10
HIV antigen/antibody	+	Negative	SS-B antibody (U/mL)	<1.0	<10
HIV-1 RNA PCR	–	Negative	CCP antibody (U/mL)	<0.6	<4.5
CD4 (/uL)	670	500-1400	RNP antibody	–	Negative
CD8 (/uL)	211	200-900	Sm antibody	–	Negative
CD4/CD8	3.1	1.0-2.0	Scl-70 antibody (U/mL)	<0.6	<7
MPO-ANCA (U/mL)	<1.0	<3.5	Centromere antibody (U/mL)	<2.0	<10
PR3-ANCA (U/mL)	<1.0	<3.5	Pol III antibody (Index)	<5.0	<27.9

## Discussion

DAH is a rare but severe complication of *M. pneumoniae* infection that can occur not only in immunocompromised patients but also in immunocompetent individuals [5-9]. *M. pneumoniae* infection complicated by DAH often presents with multi-organ involvement, including hepatitis and bone marrow suppression [5]. Our patient similarly developed liver dysfunction, coagulopathy, and deep vein thrombosis. Treatment typically consists of appropriate antibiotics combined with corticosteroids, and in some cases, plasma exchange therapy may be considered [5]. The dramatic clinical improvement observed after initiating combination therapy with minocycline and corticosteroids suggests a potentially crucial role for corticosteroids in managing DAH associated with *M. pneumoniae* infection. While direct evidence for corticosteroid use in bacterial pneumonia-associated DAH is limited, recent studies have demonstrated the benefits of corticosteroids in both severe bacterial pneumonia and refractory *M. pneumoniae* infections [10,11]. The rapid response in our case supports the concept that controlling both the infectious and inflammatory components may be crucial in managing severe *M. pneumoniae* infections complicated by DAH.

The LAMP assay has been developed and evaluated for rapid detection of *M. pneumoniae*. Studies have shown that LAMP is highly specific, with no cross-reactivity observed for other Mycoplasma species or respiratory pathogens [12,13]. The assay demonstrates high sensitivity, with detection limits ranging from two to six copies of *M. pneumoniae* DNA [12,14]. However, as in our case, severe *M. pneumoniae* infections may present with false-negative LAMP results. This highlights the importance of using multiple diagnostic modalities for pathogen identification. In our case, rapid diagnosis was achieved through FilmArray Respiratory Panel 2.1 analysis of BALF. Similar to a reported case of prolonged COVID-19 pneumonia after lymphoma treatment where FilmArray was negative in nasopharyngeal samples but positive in BALF [15], BALF FilmArray testing may be valuable in certain clinical scenarios.

Macrolide-resistant strains of *M. pneumoniae* are increasing, with significant geographic variation. A 2000-2020 analysis reported 63% resistance rates in Asia [16]. In our case, the patient's condition deteriorated despite prior treatment with azithromycin, suggesting macrolide resistance. For macrolide-resistant *M. pneumoniae*, tetracyclines and fluoroquinolones are considered alternative treatments [17]. A meta-analysis found that tetracyclines significantly shortened fever duration and hospital stay compared to macrolides in cases with macrolide-resistant *M. pneumoniae* [18]. A recent comprehensive systematic review and network meta-analysis of 85 studies with 7,095 patients demonstrated that both tetracyclines and quinolones remain effective treatment options, with minocycline showing the highest probability of being the most effective treatment for clinical response among all antibiotics studied [19]. Clinicians should consider the second-line treatment, particularly in cases where fever persists or chest X-rays show deterioration after 48-72 hours of macrolide treatment [17]. While there is currently no systematic data specifically on tetracycline and fluoroquinolone resistance rates globally, ongoing surveillance of resistance patterns to tetracyclines and fluoroquinolones remains critically important as these antibiotics become increasingly relied upon for treating macrolide-resistant infections.

The white plaque lesions observed in the trachea, in our case, likely represent airway lesions associated with *M. pneumoniae *infection. Similar cases have been reported, showing necrosis of the vocal cords, trachea, and bronchial mucosa, accompanied by large amounts of white, viscous sputum within the bronchial lumen [20]. Pathological examination of the bronchial mucosa in such cases has typically revealed evidence of inflammatory necrosis [20].

One limitation of this case is that we cannot completely exclude the possibility of initial pneumonia caused by a different pathogen followed by a secondary *M. pneumoniae *infection. Although several features supported *M. pneumoniae* as the primary pathogen, including the gradual clinical progression and significantly elevated *M. pneumoniae* PA titer, the complex nature of respiratory infections means that co-infection or sequential infection scenarios remain possible. This highlights the importance of maintaining a broad diagnostic perspective when managing complex pneumonia cases, particularly those that show an unusual clinical course or incomplete response to initial therapy. Similar cases in the future might benefit from more comprehensive microbiological testing early in the disease course to better characterize potential co-infections or sequential infections. Another limitation is the lack of antimicrobial susceptibility testing. While the patient's clinical course was consistent with macrolide-refractory disease, we cannot definitively confirm macrolide resistance without in vitro susceptibility testing. The inability to perform susceptibility testing due to current laboratory constraints in Japan highlights the challenges in clinical decision-making and the need for greater availability of *M. pneumoniae* susceptibility testing capabilities.

## Conclusions

This case demonstrates that *M. pneumoniae* infection can cause serious complications, including DAH, even in immunocompetent patients. Our experience demonstrates the value of lower respiratory tract sampling, as FilmArray analysis of BALF enabled the successful detection of *M. pneumoniae* when throat LAMP testing was negative, emphasizing the crucial role of sampling site selection in diagnosing severe respiratory infections. When faced with antibiotic-refractory pneumonia presenting with bilateral infiltrates and DAH, clinicians should consider *M. pneumoniae* infection and pursue appropriate diagnostic sampling coupled with targeted antibiotic therapy and corticosteroids for optimal outcomes.
